# Mitigation of Atherosclerotic Vascular Damage and Cognitive Improvement Through Mesenchymal Stem Cells in an Alzheimer’s Disease Mouse Model

**DOI:** 10.3390/ijms252313210

**Published:** 2024-12-09

**Authors:** Woong Jin Lee, Kyoung Joo Cho, Gyung Whan Kim

**Affiliations:** 1 Department of Neurology, College of Medicine, Yonsei University, Seoul 03722, Republic of Korea; osang0616@yuhs.ac; 2 Department of Life Science, Kyonggi University, Suwon 16227, Republic of Korea; kcho0611@kgu.ac.kr

**Keywords:** cognitive impairment, vascular dementia, Alzheimer’s disease, mesenchymal stem cell, high-fat diet

## Abstract

Alzheimer’s disease (AD) is a neurodegenerative condition characterized by progressive memory loss and other cognitive disturbances. Patients with AD can be vulnerable to vascular damage, and damaged vessels can lead to cognitive impairment. Mesenchymal stem cell (MSC) treatment has shown potential in ameliorating AD pathogenesis, but its effect on vascular function remains unclear. This study aimed to improve cognitive function by alleviating atherosclerosis-induced vessel damage using MSCs in mice with a genetic AD background. In this study, a 5xFAD mouse model of AD was used, and atherosclerotic vessel damage was induced by high-fat diets (HFDs). MSCs were injected into the tail vein along with mannitol in 5xFAD mice on an HFD. MSCs were detected in the brain, and vascular damage was improved following MSC treatment. Behavioral tests showed that MSCs enhanced cognitive function, as measured by the Y-maze and passive avoidance tests. Additionally, muscle strength measured by the rotarod test was also increased by MSCs in AD mice with vessel damage induced by HFDs. Overall, our results suggest that stem cells can alleviate vascular damage caused by metabolic diseases, including HFDs, and vascular disease in individuals carrying the AD gene. Consequently, this alleviates cognitive decline related to vascular dementia symptoms.

## 1. Introduction

Increased lifespan has resulted in a rise in geriatric diseases, particularly brain dysfunction. Among these, cognitive dysfunction, known as dementia, is the most prevalent and is associated with degenerative mental disorders. The symptoms of dementia primarily manifest as cognitive impairment, which can significantly interfere with daily life [1]. Cognitive impairment is generally classified as being derived from either Alzheimer’s disease (AD) or vascular disease (vascular dementia, VaD) [2]. AD is a neurodegenerative condition that occurs when amyloid β-peptide (Aβ) is deposited in brain neurons, creating blockages between synapses, leading to the loss of synaptic connections or the formation of neurofibrillary tangles [3]. VaD, on the other hand, is caused by brain damage resulting from cerebrovascular disease, such as ischemia, thrombosis, or cerebral hemorrhage [4], which also leads to cognitive dysfunction. Cerebrovascular diseases such as thrombosis, hypoxia, and vascular lesions, along with risk factors such as hypertension, atherosclerosis, diabetes, smoking, and alcoholism, are major etiological factors for dementia [1,5]. Aβ-related disorders, such as AD, are known to be susceptible to vascular damage, which can lead to impaired vascular function and potentially contribute to cognitive impairment. High-fat diets (HFDs) can actually accelerate AD pathology and progression [5,6].

Vascular impairments and AD are closely linked to vascular dysfunction, contributing significantly to the onset and progression of AD [7]. This interaction is evident in neurovascular unit dysfunction [8,9], including vessel disease [10,11] and impaired amyloid-beta clearing [12], and risk factors associated with aging [13]. In AD, the neurovascular unit, which consists of neurons, glial cells, and blood vessels, is dysfunctional [8]. These vascular abnormalities can exacerbate Aβ accumulation, a hallmark of AD pathology [12]. Vascular impairments can hinder the clearance of amyloid-beta from the brain. Disruptions in the blood–brain barrier may lead to amyloid-beta accumulation, contributing to AD pathology [13]. Metabolic disorders such as hypertension, diabetes, and hyperlipidemia are common risk factors for both vascular impairments and AD. These conditions can lead to vascular damage, which in turn may promote or exacerbate AD pathology [14]. Managing these risk factors is crucial in reducing the risk of both vascular cognitive impairment and AD. Further understanding the interplay between vascular impairments and AD is essential for developing comprehensive strategies to prevent and treat cognitive decline. A study of 5xFAD mice on an HFD showed that circulating lipids increased, and glucose intolerance worsened [15]. Many recent studies indicate that metabolic dysfunction, including increased neuroinflammation, is associated with the main pathology of cognitive impairment [16,17,18]. Vascular damage from HFD is prone to forming atheroma in the carotid arteries. Metabolic dysfunction, along with vascular damage, may rapidly exacerbate cognitive impairment and hasten the decline of cognitive function in individuals with a genetic predisposition. Post-mortem examinations of patients who died from Alzheimer’s dementia have revealed significant brain tissue abnormalities indicative of VaD [19].

Mesenchymal stem cells (MSCs) exist in various tissues and form colonies in experimental environments. They are characterized by their ability to differentiate into various tissues of mesodermal origin, including adipose, cartilage, bone, and nerve cells. Blood-derived MSCs have been proposed as new stem cells that can replace other sources, such as fat cells and bone marrow [20]. MSCs have been shown to provide a protective role in atherosclerosis [21], due to their anti-inflammatory properties, particularly in animal models of atherosclerosis [22]. MSC transplantation provides a beneficial cellular environment by modulating cytokine and chemokine secretion and reducing endothelial dysfunction. The pathophysiology of atherosclerosis is complicated and closely related to the inflammatory responses of vascular endothelial cells (ECs) [23]. MSCs improve endothelial function by activating endothelial nitric oxide synthase (eNOS) and subsequently mitigating atherosclerosis [24]. Stem cell therapy has recently gained attention as a potential treatment for various intractable diseases, including neurodegenerative diseases [25]. While MSC treatment has shown promise in mitigating the progression of these disorders, its specific impact on vascular function remains unclear. Blood-derived MSCs may be useful for treating vascular diseases, such as vascular dementia and atherosclerosis, in the future [26], although several challenges remain before they can be applied in clinical practice.

To further investigate the potential of stem cells as a treatment for cognitive impairment, we created an animal model of vascular dementia in mice and evaluated the therapeutic effects of stem cells on the disease. Under AD conditions, which can be vulnerable to vascular damage and lead to vascular dysfunction, MSC treatment has shown potential to improve the pathogenesis of these disorders. However, its effect on vascular function remains unclear. This study aimed to improve impaired cognitive function by alleviating atherosclerosis-induced vessel damage using MSCs in mice with a genetic background.

## 2. Results

### 2.1. Formation of Atheroma Induced by High-Fat Diets in the Carotid of 5xFAD Mice

**Figure 1:**
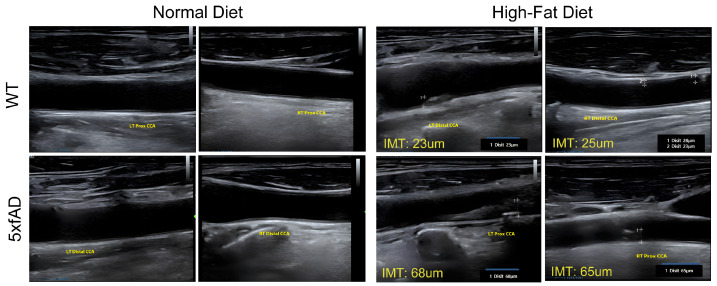
Formation of atheroma was induced by high-fat diet. Atherosclerosis was induced by high-fat diet (HFD) for 5 weeks in WT mice and amyloid β-peptide-related mice (5xFAD). Ultrasound sonography was used to examine atheroma formation in the common carotid artery (CCA). The size of the atheromatic plaque was measured at its thickest part (the white dotted line), and carotid intima–medial thickness (IMT) was presented as a number in yellow. The upper panel shows results in WT, and the lower panel in 5xFAD. Plaques formed by HFD are shown in the right panel. *n* = 5.

This study induced vascular damage using a HFD. Primarily, WT (*n* = 5 for each group) and AD mice (*n* = 8 for each group) were divided into two groups and fed either a normal chow diet (NCD) or HFD for 7 weeks. Ultrasound sonography was used to examine atheroma formation in the common carotid artery (CCA). Atheromatic plaques were detected in AD mice but not in WT mice (Figure 1). In the CCA of AD mice fed an HFD, a single plaque was found in an arbitrary region. The size of the atheroma was measured at its thickest part, and the carotid intima–medial thickness (IMT) in the HFD-fed mice group was 66 μm, more than double the 23 μm IMT in the NCD-fed group. Biochemical analysis of plasma showed that total cholesterol and low-density lipoprotein (LDL) were significantly increased in the AD-HFD group, as shown in Table 1. Although most biochemical values showed slight alterations in the AD-HFD group, there were no significant differences between the groups, except for total cholesterol, which was higher in the AD-HFD group compared to AD-NCD. Glucose levels were significantly lower in the AD-HFD group than in the AD-NCD group, possibly indicating that HFD can lead to metabolic dysregulation (see Table 1).

### 2.2. Mesenchymal Stem Cell Isolation, Identification, and Differentiation Assay

**Figure 2:**
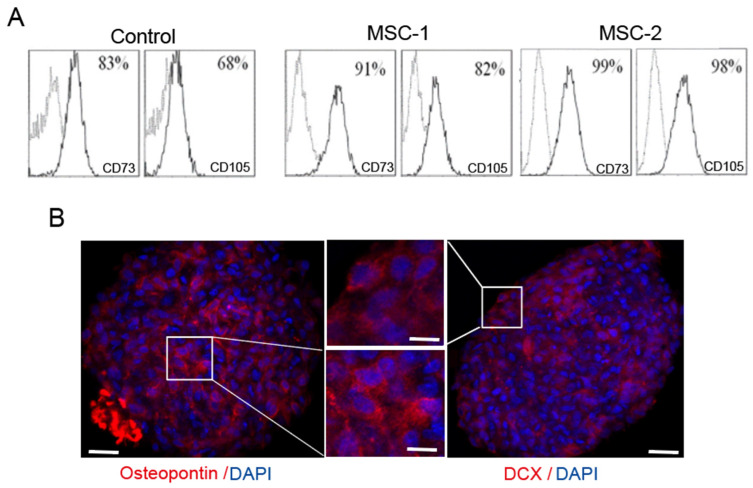
Mesenchymal stem cell isolation, identification, and differentiation. (**A**) Mesenchymal stem cells (MSCs) were characterized using flow cytometry with FITC-conjugated CD73 and CD105 antibodies as MSC markers. (**B**) MSC multipotency was verified by inducing differentiation into bone or neurons. Osteopontin-positive cells (red, left image) indicate osteoblast differentiation, and doublecortin (DCX)-positive cells (red, right image) indicate neuronal differentiation. Scale bar: 20 μm, 50 μm. *n* = 4.

MSCs have been found in almost all tissues and can be derived from them. In this study, C57BL/6 mouse bone marrow mesenchymal stem cells (MSCs) were obtained from Cyagen (Santa Clara, CA, USA) and cultured in DMEM supplemented with 20% fetal bovine serum and 1% penicillin–streptomycin. MSCs were confirmed using cell markers such as CD73 and CD105 through FACS analysis (Figure 2A). CD45 was used as a negative cell marker. Before MSC transplantation, their multipotency was verified. The cultured MSCs were induced to differentiate into osteoblasts in osteogenic or neuronal differentiation media, refreshed every 3–4 days. Osteopontin-positive cells (Figure 2B, left) and DCX-positive cells (Figure 2B, right) were successfully detected. The osteopontin- or DCX-positive signals of MSCs were scarcely detected in normal media without any differentiating factors (Supplementary Figure S2).

### 2.3. Introduced MSCs Reached the Brain

**Figure 3:**
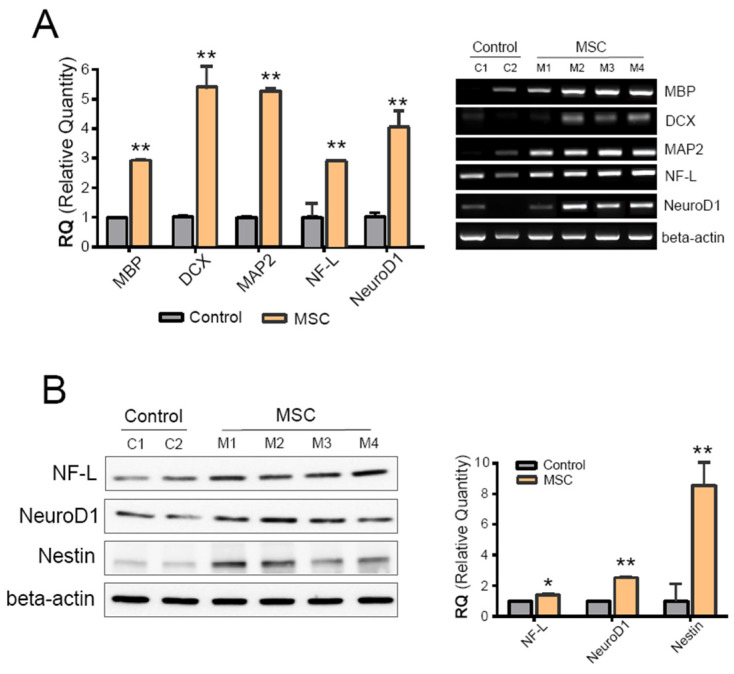
Introduction of MSCs to the brain. (**A**) RT-qPCR was performed on the brain tissue of the control group (n = 2) and MSC-treated group (*n* = 4). The level of mRNA expression of NeuroD1, MAP2, DCX, and NF-L was confirmed and is displayed in a graph (left); gel image of the final PCR product (right). (**B**) Protein expression levels of NF-L, NeuroD1, and Nestin were analyzed by Western blot. Quantitative results are shown in the right graph. The quantitative graph is the result of statistical analysis of all the results obtained through five independent WB experiments or qPCR using samples obtained from each animal. The symbol * indicates the statistical difference between marked groups (* *p* < 0.01; ** *p* < 0.01).

To deliver MSCs to damaged areas such as blood vessels and the brain, MSCs were injected into the tail vein with mannitol to loosen the blood–brain barrier (BBB) and allow MSCs to traverse into the brain (Figure S1). qPCR was performed on brain tissue to determine if MSCs had migrated to the damaged site, the blood vessels, and the brain. Additionally, the transplanted MSCs were evaluated for differentiation into neural cells. The mRNA expression of NeuroD1, microtubule-associated protein 2 (MAP2), doublecortin (DCX), and NF-L, which are nerve-specific markers, was higher in the MSC-transplanted group (*n* = 4) than in the control group (*n* = 2) (Figure 3A). MAP2 and DCX were used as markers of neural growth and axonal regeneration, respectively. The relative quantity of expressed transcripts was shown (Figure 3A, left), and the final products were visualized on agarose (Figure 3A, right). Western blot results showed protein expression levels of NF-L, NeuroD1, and Nestin from the hemisphere of the mouse brain (Figure 3B, left image). These proteins are involved in neuronal differentiation and regeneration. The expression levels of these neural marker proteins were higher in the MSC-treated group (*n* = 4) compared to the control group without MSC treatment (*n* = 2). We quantified the amount of protein expression and presented it in a graph (Figure 3B, right graph), including NF-L (neurofilament light polypeptide), NeuroD1 (neuronal differentiation 1), and Nestin, an intermediate filament involved in neuronal growth, particularly in axons.

### 2.4. MSCs Mitigated Vascular Damage in AD Mice with HFD

**Figure 4:**
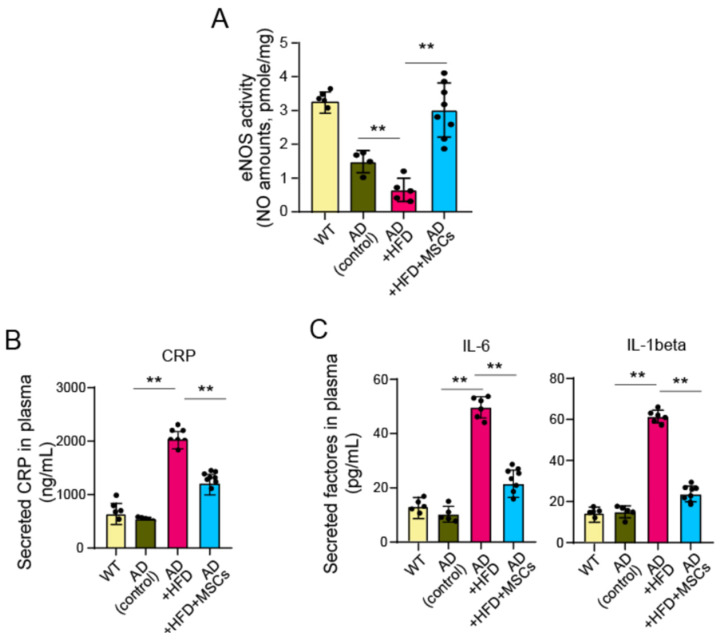
Vascular damage in AD mice fed HFDs and the effect of MSCs. (**A**) The effect of implanted MSCs on damaged vessels with atheroma formation was measured by eNOS activity. NO in tissue lysates was measured, and eNOS activity was calculated based on nitrite concentration from the standard curve. The animal number used in each group: WT, *n* = 5; AD, *n* = 5; HFD, *n* = 6; HFD + MSC, *n* = 8. (**B**) CRP (*n* = 5) and inflammatory cytokines IL-6 (*n* = 5) and IL-1β (*n* = 5) (**C**) were quantified in total serum using colorimetric ELISA. NCD, normal chow diet; HFD, high-fat diet. WT, wild-type; AD, Alzheimer’s disease. The symbol * indicates a statistical difference between marked groups (** *p* < 0.01).

It was confirmed that MSCs injected into the tail vein migrated successfully to the mouse brain and differentiated into neurons. MSCs were administered for a relatively short-term period of 14 weeks in 6-week-old AD mice, which is relatively early, similar to previously reported studies [19]. To investigate changes related to vascular condition, eNOS expression was examined in the carotid artery of MSC-treated AD-HFD mice (Figure 4A). MSC-treated AD mice showed a clear recovery of eNOS expression, even under HFD conditions. There was a marked difference in inflammation-related changes in the MSC-treated AD-HFD group, with a significant improvement in damaged blood vessels. This is thought to be due to changes in inflammatory factors induced by MSCs. To measure inflammation in 5xFAD mice fed HFDs and treated with MSCs, plasma was analyzed using ELISA. IL-1β, IL-6, and CRP, key inflammatory components, were significantly increased in AD mice fed HFDs. Our results showed that the inflammatory condition was mitigated in the MSC-treated AD-HFD group (Figure 4B). IL-1β and IL-6 are pro-inflammatory cytokines that play pivotal roles in initiating and amplifying inflammatory responses. These cytokines contribute to CRP production in the liver, which is widely used as an inflammatory marker.

### 2.5. MSCs Improved Cognitive Function in AD Mice with HFD

**Figure 5:**
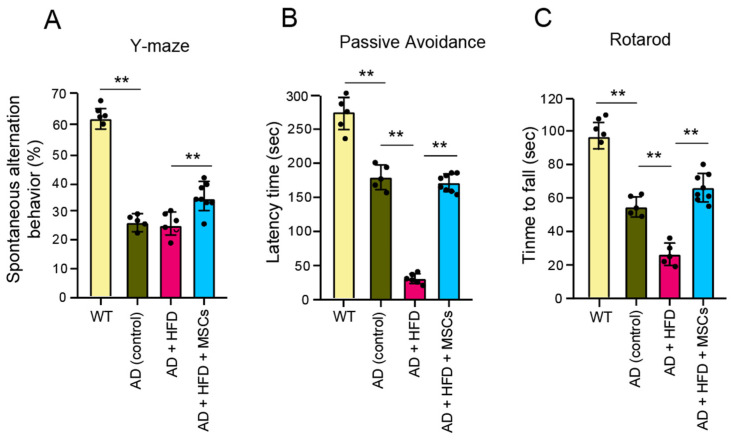
Effect of MSC treatment on cognitive function. (**A**) The Y-maze behavior test was performed to evaluate spatial cognition as a measure of short-term memory. Entries were only recorded if the tail completely entered the branch. (**B**) The passive avoidance test was conducted to measure working memory ability (LeDoux’s method). The time taken by the mouse to enter the dark side after the guillotine door was opened was measured. Latency time was recorded when all four feet of the mouse entered the dark side. (**C**) The rotarod test was performed using the acceleration rotation method, starting at 0 rpm and accelerating to 25 rpm over 2 min. The animal number used in each group (** *p* < 0.01): WT, *n* = 5; con, *n* = 5; HFD, *n* = 6; HFD + MSC, *n* = 8.

The Y-maze experiment showed a statistically significant decrease in performance for the AD animal models group compared to the control AD-NCD group (Figure 5A). However, the MSC-treated AD-HFD group showed a tendency toward behavioral recovery, such that there was no significant difference from the control group. In the passive avoidance test, AD mice demonstrated a decrease in fear-conditioning memory compared to the control AD-NCD group, and this difference was statistically significant (Figure 5B). However, the MSC-treated group showed a tendency to recover behavioral performance to the point where there was no significant difference from the control group. The blood-derived MSCs largely offset the negative effects of VaD. Both the control and MSC-treated groups showed improved performance over repeated trials, as indicated by an increasing latency to fall in the rotarod test. However, a significant increase in latency to fall was observed starting on day 10 in the MSC-treated AD-HFD group, while only a small increase was seen in the control AD-NCD group (Figure 5C). This suggests that the potential for motor performance improvement was greater in the MSC-treated group.

## 3. Discussion

VaD has a high incidence after stroke or transient cerebral ischemia, but persistent cerebral blood flow reduction is also a major cause of dementia. VaD causes significant burdens to patients and their families, though it can be highly preventable if the primary disease onset is suppressed. This study showed that MSCs have the potential to prevent and treat dementia, which has been partially verified.

In our study, AD mice fed with HFD showed more vulnerability to vascular damage by forming atheromas (Figure 1) and exhibited significant cognitive decline in the Y-maze test (Figure 5). MSC treatment improved the declined cognitive function (Figure 5). Although MSCs did not increase the number of neurons in AD animal models or directly remove or reduce atheromas, the implanted MSCs had a beneficial effect by increasing eNOS levels (Figure 4). The alleviation of vascular damage by MSCs occurred through their paracrine effect rather than by directly restoring neurons. Consequently, cognitive decline was improved. A difference between the MSC-treated AD-HFD and AD-HFD groups was observed in the eNOS and ELISA assessments of inflammatory markers in CCA and whole blood plasma, demonstrating a positive effect of MSCs (Figure 4). This suggests that MSCs have a potential role in mitigating the negative effects of vascular dementia, especially in the vessels affected by HFD in the AD model.

MSC-treated groups showed improved motor function in the rotarod test. AD mice treated with MSCs displayed increasing latency to fall from the rotarod over subsequent trials, even though they were fed HFDs. Both the control and MSC-treated AD-HFD groups started at a similar baseline, but the potential for improvement was greater in the MSC-treated group. Motor performance and retention might be improved by MSCs. The results of the behavioral tests indicated that blood-derived stem cells have the potential to mitigate the negative effects of vascular dementia.

Most biochemical indices of the blood worsened, though not significantly, with HFD, except for glucose levels, which were significantly lower in AD mice fed HFDs (Table 1). This can be interpreted as a sign of metabolic disorders. Many studies show that dementia increases with age, especially in those with metabolic disorders such as diabetes, obesity, and a high-cholesterol diet. Additionally, brain metabolic abnormalities have been observed in younger patients with dementia through brain metabolic imaging studies. Metabolic abnormalities may cause dementia before amyloid accumulation occurs over decades [27]. Recent PET studies of young people, patients with dementia, and normal elderly people support the metabolic hypothesis of AD and the importance of the Warburg effect [28]. It has been revealed that areas of the brain in young people that are active during rest mainly use aerobic glycolysis to metabolize energy, which corresponds with the areas where amyloid accumulation begins in the elderly. The reduced glucose level observed may be related to the Warburg effect.

Although MSCs were administered for just 14 weeks and successfully delivered to the brain through the blood vessels, the AD mice treated with MSCs did not show dramatically improved performance in behavioral tests. In the passive avoidance test, statistically significant results were obtained, supporting the hypothesis of this study. However, these results were slightly weaker compared to the Y-maze results, which mainly measure cognitive and memory abilities associated with the frontal lobe and hippocampus. This might be due to the nature of the passive avoidance test, which directly reflects fear conditioning, and the relatively lesser effect of dementia on cognitive and memory abilities mediated by the amygdala in this animal model. Recent reports suggest that MSCs that have migrated to the brain can remove damaged or dysfunctional neurons [29]. As a result, cognitive functions can recover as synapses regenerate and reconnect to other neurons [30]. Furthermore, growth factors secreted by MSCs may stimulate synapse formation, and autophagy is expected to be activated [31]. When dysfunctional neurons are removed, normal brain cells may expand their networks to perform cognitive functions [32]. Thus, this approach may not be as effective in mice with severe cognitive impairment. However, in patients with mild cognitive impairment (MCI), where neurons survive but synapse connections are damaged, MSC treatment may improve cognitive function. Additionally, methods to differentiate stem cells into neurons in the brain could pose a risk of becoming cancerous, which is a major hurdle in using stem cell therapy for brain conditions. In this study, translocated MSCs were detected by MAP2 and DCX, which were used as markers of neural growth and axonal regeneration. Protein expression levels of NF-L, NeuroD1, and Nestin are involved in neuronal differentiation and regeneration. NF-L was used for neurofilament light polypeptides, NeuroD1 was used for neuronal differentiation, and Nestin represents an intermediate filament involved in neuronal growth, particularly in axons. These protein levels of the markers increased in the MSC-treated group (Figure 3), which may indicate that MSCs play a role in neuronal enhancement. However, the paracrine effect of MSCs may offer a safer and more effective application for patients with MCI.

A key mechanism by which MSCs exert their therapeutic effects is through their paracrine activity [33]. The release of bioactive molecules that modulate the surrounding environment, such as growth factors [34], cytokines [35], exosomes [36], and other signaling mediators [37], plays a pivotal role in influencing neuronal survival, reducing inflammation, and promoting neurogenesis [38]. MSCs secrete various anti-inflammatory cytokines such as IL-10 and TGF-β, which can help reduce neuroinflammation. These molecules can inhibit the activation of microglia and astrocytes, the brain’s resident immune cells, thereby reducing chronic inflammation that contributes to neuronal damage [39]. MSCs have been shown to enhance BBB integrity by releasing factors like VEGF, HGF, and MMPs (matrix metalloproteinases), which can help restore endothelial cell function and reduce the permeability of the BBB [40]. This is important for preventing further neurodegeneration and ensuring that therapeutic agents can reach the brain effectively. In addition, there is evidence suggesting that MSCs can help reduce Aβ deposition by secreting factors that modulate the activity of enzymes involved in amyloid processing [41]. MSCs may enhance the clearance of amyloid plaques by promoting the activity of microglia, which can phagocytize Aβ [42]. While much of this research is still in preclinical or early clinical stages, the potential for MSC-based therapies, especially MSC-derived exosomes, offers hope for future treatments that could slow or even reverse the progression of dementia. In addition, this study was conducted using male mice, and it is possible that gender differences may influence the outcomes. As such, the effects of MSCs on alleviating cognitive decline in female mice with AD may differ from the results observed in this study. Further research incorporating both genders is warranted to address this limitation and provide a more comprehensive understanding of MSC efficacy in AD models.

This study could not demonstrate the extent of brain cell restoration or the improvement of synaptic connections. As mentioned in the hypothesis of this study, atherosclerosis caused by aging and metabolic disturbance may lead to vascular damage, and MSCs could improve the local inflammatory environment in the blood vessels, leading to an improvement in impaired cognitive function. Even in mice with a genetic predisposition to AD, MSCs may prevent the rapid decline of cognitive function by mitigating vascular damage.

## 4. Materials and Methods

### 4.1. Animals

The animal model used in this study consisted of amyloid β-peptide-related male 5xFAD (mouse line Tg6799, AD) heterozygous mice and their littermates (C57BL/6J, WT), obtained from the Jackson Laboratory. The mice underwent a 1-week adaptation period, during which they were kept in controlled conditions: a constant temperature of 22 °C, 50–60% humidity, and a 12 h dark/light cycle. All procedures for treatment and euthanasia were reviewed and approved by the Yonsei University IACUC in accordance with the guidelines of the American Association for Laboratory Animal Science. Efforts were made to minimize animal discomfort and reduce the number of animals used. Following the adaptation period, the WT and AD mice were randomly divided into two groups: one receiving a normal chow diet (NCD) and the other a high-fat diet (HFD). Each group consisted of five to eight mice, and they were fed an NCD or HFD for 7 weeks. MSCs treatment was administered for a total of 14 weeks, starting at week 6, with MSCs injected twice—at week 6 and again at week 13.

### 4.2. Biochemical Analysis

Blood biochemical analysis was conducted in each animal group. The levels of total bilirubin, high-density lipoprotein (HDL), low-density lipoprotein (LDL), and triglycerides (TGs) were measured using commercial kits from Wako Pure Chemical Industries (Osaka, Japan). The amount of glucose in the blood was measured using a glucose check kit for diabetes.

### 4.3. BM-MSC Characterization

Mouse mesenchymal stem cells are generally isolated from an aspirate of BM harvested from the tibia and femoral marrow compartments. BM-derived MSCs were purchased from Cyagen (cat # MUBMX-01001, Santa Clara, CA, USA). Cells were cultured in Dulbecco’s modified Eagle’s medium (DMEM, Gibco, Waltham, MA, USA) containing fetal bovine serum (FBS, Gibco, Waltham, MA, USA), 1% penicillin–streptomycin, and valproic acid (Sigma, St. Louis, MJ, USA) in a 37 °C, 5% CO_2_ incubator. Cells were characterized using flow cytometry analysis and confirmed to be MSCs by the standard MSC markers CD73 (BD Biosciences, San Jose, CA, USA) and CD105 (BD Biosciences, San Jose, CA, USA). The cells were enzymatically dissociated into mononuclear cells using a 0.25% trypsin/EDTA solution. These cells were subsequently washed twice with Hank’s Balanced Salt Solution (HF2, Invitrogen, Carlsbad, CA, USA) and supplemented with 2% FBS (Gibco, Waltham, MA, USA). The cells were then transferred to a 1.5 mL tube and treated with a 1:100 dilution of fluorescein isothiocyanate (FITC)-labeled CD73 and CD105 antibody solution (BD Biosciences, San Jose, CA, USA). The reaction was carried out at 4 °C for 1 h. Subsequently, the cells were washed twice with HF2, and the pellet obtained after centrifugation was collected and resuspended in a flow cytometry solution containing propidium iodide (1 μg/mL, Sigma, St. Louis, MJ, USA), which serves as a marker for dead cells. The samples were kept on ice until analysis. Stained cells were analyzed using a FACSCaliburTM Flow Cytometer (BD Biosciences, San Jose, CA, USA). MSCs were passed through a cell strainer to avoid clumping, and each injection contained 1 × 10^6^ MSCs diluted in 200 μL of sterile saline (0.9% NaCl). MSCs were administered intravenously via the tail vein with 0.5 mL of 25% mannitol (Sigma, St. Louis, MJ, USA) to allow the BBB to be penetrated. Mannitol was injected intra-peritoneally.

### 4.4. BM-MSC Multipotency Test

To confirm whether the MSCs to be used in this study had multipotency, an MSC identification kit (R&D, Minneapolis, MN, USA) was used and the test was performed according to the manufacturer’s protocol. To assess the ability of MSCs to differentiate into specific cells, MSCs were cultured in differential media for bone or neurons supplied by the differentiation kit (R&D, Minneapolis, MN, USA) according to the manufacturer’s protocol. MSCs were cultured in osteogenesis media containing supplements supplied by the kit. To validate osteogenic differentiation, the MSC culture media were replaced with osteogenic differentiation media and renewed media every 3~4 days. After 4 weeks, the differentiated cells were fixed and detected with immunocytochemistry of osteopontin (Abcam, Cambridge, MA, USA) antibody for bone or DCX antibody (SCBT, Dallas, TX, USA) for neurons.

### 4.5. Measurement of eNOS Expression in Vascular Tissue

After sacrificing the mouse, the carotid artery was removed, washed in ice-cold PBS, and homogenized with ice-cold RIPA lysis buffer. The lysates were used to measure protein concentration. The same amount of lysate was reacted with L-arginine (1 mM) as a substrate for eNOS to a final 100 uL volume in a plate. After incubation at 37 °C for 1 h, NO’s absorbance was measured at OD 540 nm by adding Griess reagent, which was additionally incubated for 10 min. The calculated eNOS activity was based on the nitrite concentration from the standard curve.

### 4.6. Protein Isolation and Western Blot Analysis

Brain tissues were collected and lysed in M-per (Invitrogen, Carlsbad, CA, USA) with protease/phosphatase inhibitor, Halt (Invitrogen, Carlsbad, CA, USA) to extract proteins. The extracted proteins were quantified by the BSA (Pierce, Waltham, MA, USA) method and separated by electrophoresis using a 10% sodium dodecyl sulfate–polyacrylamide gel (SDS-PAGE). After electrophoresis, the proteins in the gel were transferred to a nitrocellulose membrane prepared in Tris-buffered saline [TBS: 10 mM Tris-HCl (pH 7.4), 150 mM NaCl] with 0.1% Tween-20 (TBS-T) solution containing 5% (*w*/*v*) non-fat dry milk. Blocking was performed to prevent non-specific binding by soaking for 1 h with skim milk. The primary antibody reacted with membranes at room temperature (RT) for 1 h and the used primary antibodies were as follows: NF-L (1:500, R&D, Minneapolis, MN, USA), NeuroD1 (1:1000, abcam, Cambridge, MA, USA), and Nestin (1:1000, SCBT, Dallas, TX, USA). Then, the membrane was reacted with horseradish peroxide (HRP)-conjugated anti-mouse or -goat IgG antibodies (Jackson ImmunoResearch, West Grove, PA, USA) diluted in TBS-T at a ratio of 1:5000 at room temperature, washed thoroughly with TBS-T, and measured for expression using an enhanced chemiluminescence system (ECL, Cytiva, Marlborough, MA, USA). The results were quantified.

### 4.7. Real-Time Reverse Transcription Polymerase Chain Reaction (qPCR) Analysis

Total cellular RNA of the brain tissue was isolated using a HiGene Total RNA prep kit (BIOFACT Co. Ltd., Daejeon, Republic of Korea) according to the manufacturer’s instructions. Using a One-Step SYBR PrimeScript RT-PCR kit (TaKaRa Bio Inc., Shiga, Japan), RT-qPCR was performed on a QS3 real-time PCR system (Applied Biosystems, Waltham, MA, USA). According to the manual, PCR amplification was performed for 40 cycles with denaturation at 95 °C for 5 s, and annealing and extension at 60 °C for 60 s. Each sample was assessed in triplicates including a no-template control. PCR amplification was performed for NeuroD1, MAP2, DCX, and NF-L. Each amplified PCR product was normalized with a beta-actin PCR product. Each amplified gene product was compared to the corresponding gene product in the control group, and the relative values were calculated for each control. Differential expression levels were analyzed using the 2^–∆∆Ct^ method and expressed as relative quantity (RQ). The primer sequences for each gene are shown in Table 2. Additionally, the final reaction samples were loaded onto an agarose gel to visualize the amplified gene product.

### 4.8. Sonography

The mouse carotid artery ultrasound examination system consists of the following: a VisualSonics Vevo 2100 system (FUJIFILM VisualSonics, Toronto, ON, Canada), a 30 MHz ultrasound probe, a mouse body frame, and a mouse respiratory control unit. Prior to the mouse carotid artery ultrasound examination, the following procedures were performed. Noradrenaline (0.01 mg/g) was administered to the mouse to calm it down. The mouse was fixed onto the mouse body frame. The mouse was connected to a mouse respiratory control unit, which maintained a temperature of 37 °C. The mouse carotid artery ultrasound examination was conducted by placing the mouse onto the ultrasound probe and mouse body frame. Ultrasound gel was applied to the mouse carotid artery area, and the ultrasound probe was positioned on top of the gel. The VisualSonics Vevo 2100 system software was used to obtain B-mode images. Color Doppler mode was used to identify the inner and outer walls of the carotid artery and obtain images. Pulsed-wave Doppler mode was used to analyze the blood flow state inside the carotid artery and obtain images.

### 4.9. Analysis of Inflammatory Factors by ELISA

The levels of inflammatory cytokines IL-6, IL-1β, and CRP were quantified in total blood plasma using a colorimetric ELISA kit (KHB3441, Invitrogen, Carlsbad, CA, USA) according to the manufacturer’s instruction. The results were expressed as mean values and standard deviations, and the statistical processing was performed using SPSS for Windows version 21.0 (SPSS Inc., Chicago, IL, USA). The Mann–Whitney U test was used as a non-parametric method to compare the difference between the experimental groups, and a *p*-value of 0.05 or less was considered statistically significant.

### 4.10. Behavioral Tests

The Y-maze experiment was conducted to assess spatial cognition in the form of short-term memory. The Y-maze apparatus consists of three arms. Each mouse was placed on one of the arms and allowed to explore freely for 8 min. The entries into each arm were recorded only if the mouse’s tail fully entered the branch. Points were awarded if the mouse entered three different branches consecutively.

The passive avoidance test was used to evaluate the working memory of rodents, a standard method to measure learning and memory. The experiment followed LeDoux’s method. One hour before the experiment, the mouse was moved to the behavioral observation room. In the training trial, the light in the right room of the shuttle box was turned on, and the mouse was gently placed in the box, facing away from the guillotine door. After 10 s of exploring the box, the guillotine door was opened, and the mouse instinctively moved to the dark left room. Mice that did not enter the dark side within 40 s were excluded. Upon entering the dark side, the guillotine door closed, and a 0.5 mA electrical shock was applied through the grid floor for 3 s, which the mouse remembered. The latency time—the time taken by the mouse to re-enter the dark side after the guillotine door opened—was measured. A test trial was conducted 24 h after the training trial to assess the effects of drug administration on long-term memory. The time taken for the mouse to enter the dark side (latency) was recorded, with a maximum limit of 300 s.

The rotarod test was conducted using a treadmill acceleration method, beginning at 0 rpm and increasing to 25 rpm over 2 min. The mouse was placed in the center of the rotating rod, and the time it took to fall off was recorded. Each animal was tested five times, with the average of the three middle values (excluding the highest and lowest) used as the final result. This experiment was conducted daily over a 14-day period.

### 4.11. Statistical Analysis

In the animal behavior test for evaluating cognitive function, the statistical analysis was conducted using the SPSS for Windows version 21.0 (SPSS Inc, Chicago, IL, USA) program. The test results were presented as mean values and standard deviations. A non-parametric analysis, one-way ANOVA, was used to evaluate the differences among the experimental groups for the results shown in Figure 4 and Figure 5 (Bartlet’s test). A *p*-value of 0.05 or less was considered statistically significant. The number of animals used in all experiments was *n* = 4–8 mice per group. For the statistical significance of biochemical analysis shown in Figure 3, data are expressed as mean ± SEM. The statistical analysis of the results in Figure 3 used a parametric method, the *t*-test. Differences were indicated with asterisks for statistical significance at * *p*< 0.01 and ** *p*< 0.001.

## Figures and Tables

**Table 1:** Biochemical analysis in plasma.

	Wild-Type		5XfAD	
	Normal Chaw Diet	High-Fat Diet	Normal Chaw Diet	High-Fat Diet
Body weight (g)	25.0 ± 1.5	32.0 ± 2.0 *	23.8 ± 1.6	25.0 ± 2.9
T-blirubine (mg/dL)	0.35 ± 0.1	0.52 ± 0.1	0.4 ± 0.1	0.5 ± 0.1
T-chol (mg/dL)	152.1 ± 11.0	251.7 ± 17.6 *	170.0 ± 2.5	193.2 ± 10.3
TG (mg/dL)	106.3 ± 12.8	211.0 ± 26.7 *	96 ± 2.7	93.6 ± 3.1
LDL (mg/dL)	51.4 ± 5.1	122.3 ± 9.8 *	58 ± 1.5	70.3 ± 2.8 *
HDL (mg/dL)	45.3 ± 5.1	33.1 ± 4.5	47.5 ± 1.8	48.8 ± 1.7
Glucose (mg/dL)	91.0 ± 8.7	128.2 ± 13.6	88.43 ± 5.1	58.1 ± 6.4 *

* The symbol (*) indicates a statistical difference between marked groups.

**Table 2:** Primer sequences for qPCR.

Gene	Primer Sequences	
	Forward	Reverse
*DCX*	5′-CTGACTCAGGTAACGACCAAGAC-3′	5′-TTCCAGGGCTTGTGGGTGTAGA-3′
*NF-L*	5′-GCCTTGGACATCGAGATTGCAG-3′	5′-CAAGCCACTGTAAGCAGAACGG-3′
*NeuroD1*	5′-CCTTGCTACTCCAAGACCCAGA-3′	5′-TTGCAGAGCGTCTGTACGAAGG-3′
*MAP2*	5′-GCTGTAGCAGTCCTGAAAGGTG-3′	5′-CTTCCTCCACTGTGGCTGTTTG-3′
*beta-actin*	5′-CCTGAACCCTAAGGCCAACC-3′	5′-ATGGCGTGAGGGAGAGCATA-3′

## Data Availability

Data is contained within the article and Supplementary Materials.

## Supplementary Materials

The following supporting information can be downloaded at https://www.mdpi.com/article/10.3390/ijms252313210/s1.
