# Supplementary material for: Mitigation of Atherosclerotic Vascular Damage and Cognitive Improvement Through Mesenchymal Stem Cells in an Alzheimer’s Disease Mouse Model
Source: Int J Mol Sci. 2024 Dec 9;25(23):13210. doi: 10.3390/ijms252313210 (PMC11642414; doi:10.3390/ijms252313210)
Supplement: Supplementary file 1 [file ijms-25-13210-s001.zip › ijms-3303423-supplementary.pdf]

Supplementary Data

Title:

Mitigation of Atherosclerotic Vascular Damage and Cognitive Improvement through Mesenchymal Stem Cells in an Alzheimer's Disease Mouse Model

Authors:

Woong Jin Lee, Kyoungjoo Cho, and Gyung Whan Kim

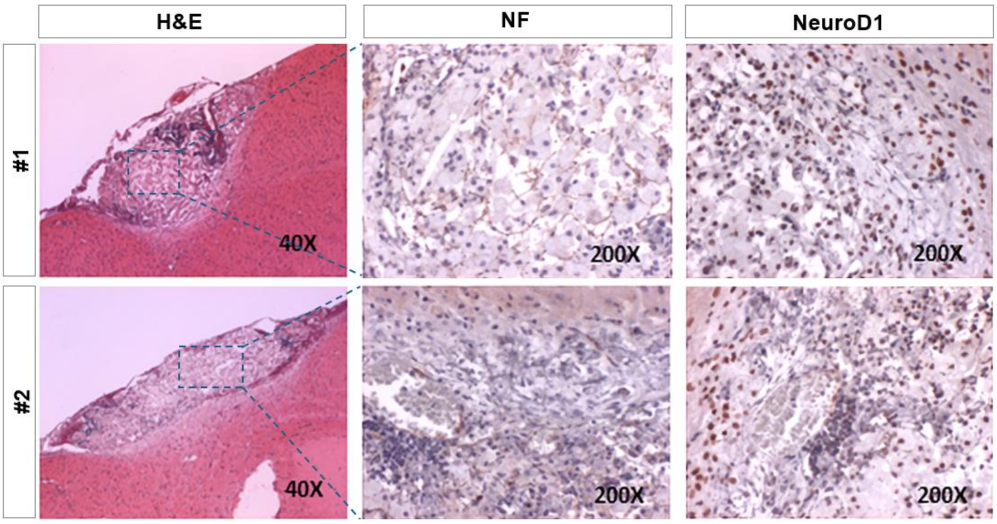

Supplementary Figure S1. Located neuronal cells in AD mice injected MSCs via tail vein (n=2). H&E staining showed the MSCs in the brain parenchyma region (the left), and immunohistochemistry of Neurofilament (NF, the middle) and NeuroD1 (the right).

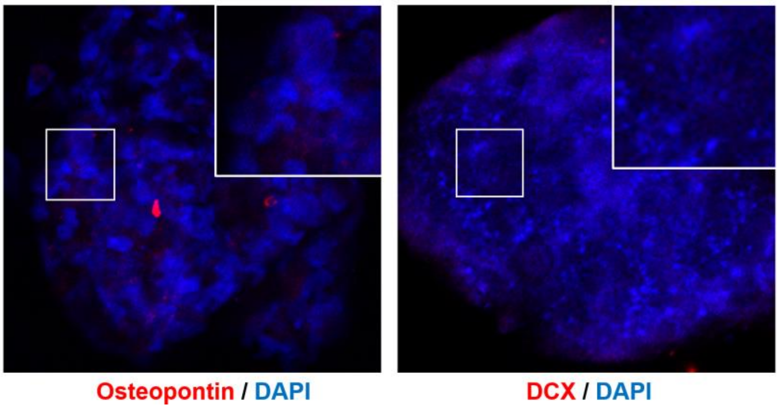

Supplementary Figure S2. The osteopontin or DCX-positive cells under condition of non-differentiation. The osteopontin or DCX-positive signals were scarcely detected in normal media that is composed without any differentiating factors.
